# Impact of China’s Drug Regulatory Reforms on the Development of Anti-Tumor Drugs

**DOI:** 10.34133/cancomm.0032

**Published:** 2026-06-04

**Authors:** Ling Tang, Lijia Yuan, Ying Geng, Zhimin Yang, Guo Huang

**Affiliations:** ^1^ Center for Drug Evaluation, National Medical Products Administration, Beijing, P. R. China.; ^2^ National Medical Products Administration, Beijing, P. R. China.

## Background

Since the launch of the 14th Five-Year Plan period (2021 to 2025), China has been implementing its innovation-driven development strategy and the Healthy China Initiative. The drug regulatory authorities have continuously advanced reforms of the drug review and approval system, introducing a series of measures to support innovative drug research and development (R&D). Under Chinese laws, an innovative drug is defined as a novel compound that has not yet been marketed domestically or internationally. Driven by collaborative efforts across all stakeholders, innovative drugs have become an engine and strategic pivot for the high-quality development of China’s pharmaceutical industry. In 2025, the National Medical Products Administration (NMPA) approved 76 innovative drugs, including 47 small-molecule drugs, 23 biologics, and 6 traditional Chinese medicines [1]. In the same year, the total value of license-out deals involving Chinese innovative drugs exceeded USD 130 billion [1].

The R&D of anti-tumor drugs in China has progressed rapidly with sustained and robust innovative momentum. These advances have substantially narrowed the gap between China and the global standard of care, while substantially broadening the therapeutic options available to patients. Concurrently, the paradigm of drug R&D has undergone a profound transformation. Chinese pharmaceutical companies have shifted their focus from generic drugs to innovative therapies, whereas multinational companies have transitioned from a time when the marketing approval in China lagged behind to an era characterized by global simultaneous development.

The advancement and reforms of China’s drug evaluation system have provided more scientific and standardized guidance for drug development. This article reviewed the evolution of China’s reforms in drug review and approval from multiple perspectives, with a focus on their impact on the R&D of anti-tumor drugs.

## Reforms Toward More Proactive Service

During the lengthy life cycle of drug R&D, integrating regulation with professional services is designed to mitigate the uncertainty inherent in innovation with the certainty provided by robust regulation. The NMPA has established a working model featuring proactive engagement, tailored guidance for each sponsor, end-to-end regulatory guidance, and synergy between drug development and regulatory review. This represents a shift away from the conventional model of review-after-submissions. Instead, regulatory resources were directed proactively toward areas of unmet medical needs and breakthrough innovation, establishing preferential policy pathways for the development of innovative drugs.

Under the oversight of the NMPA, the Center for Drug Evaluation (CDE) has continuously refined the operational framework of preapplication guidance, in-process collaboration, and postapplication review in practice. It has established a high-efficiency communication mechanism between drug sponsors and technical reviewers. In accordance with regulated response time frames, CDE-managed consultations are categorized into 3 types: Type I consultations within 30 working days, Type II consultations within 60 working days, and Type III consultations within 75 working days [2]. Based on data from the CDE review system, between 2021 and 2025, the CDE completed a total of 25,954 consultations, including 4,030 Type I, 14,991 Type II, and 6,933 Type III consultations. Of these, 5,996 consultations were related to anti-tumor drugs, including 1,478 pre-Investigational New Drug (IND) consultations and 1,053 end-of-Phase II consultations.

## Optimized Review and Approval Process for Innovative Research and Development

Optimized review and approval processes exert a comprehensive, profound, and systematic influence on pharmaceutical innovation. The *Provisions for Drug Registration* [3] issued by NMPA in July 2020 established 4 prioritized pathways, which are mainly designated to accelerate drug registration and marketing approval, namely, Conditional Approval, Breakthrough Therapy Designation (BTD), Priority Review, and Special Approval Procedures.

With the exception of the special approval procedures for preventive and therapeutic drugs for public health emergencies, anti-tumor drugs constituted the predominant category among recipients of the 3 accelerated pathways. As of December 2025, 154 drugs were granted conditional approval (including 137 anti-tumor indications, accounting for 88.9%), 335 drugs received BTD (including 227 anti-tumor indications, 67.8%), and 629 drugs were granted priority review (including 264 anti-tumor indications, 41.9%).

Conditional approval represents the most transformative reform among the accelerated regulatory pathways. The *Opinions on Deepening the Reform of the Review and Approval System to Encourage Innovation of Drugs and Medical Devices* [4] issued in October 2017 formally introduced the concept of conditional approval. Subsequently, the *Drug Administration Law of the People’s Republic of China* [5], revised and enacted in 2019, further established the legal framework. Furthermore, the *Provisions for Drug Registration* [3] explicitly specify the applicable scenarios for conditional approval. As of December 2025, among the 137 anti-tumor indications granted conditional approval, 36 had completed confirmatory trials and been converted to full approval. Conversely, 4 indications were revoked due to failure to complete the required confirmatory trials within the statutory timeframe, constituting noncompliance with the *Provisions for Drug Registration*. This demonstrates the stringent enforcement of China’s conditional approval regulatory framework.

The BTD targets innovative drugs or improved new drugs indicated for life-threatening conditions and diseases that severely impair patients’ quality of life, for which effective therapies are lacking. It also applies to drugs for which early clinical trial data demonstrate substantial clinical advantages over existing therapies. For sponsors, BTD confers benefits including an accelerated development timeline and enhanced regulatory interaction. By the end of December 2025, among anti-tumor drugs that had been granted BTD, 194 drugs held a total of 250 Type I consultations; 57 drugs had been approved. The median time from initial IND submission in China to the first New Drug Application (NDA) approval was 4.5 years, whereas the median time for domestically developed drugs in China was 4.9 years.

Drugs eligible for conditional approval or BTD may apply for priority review at the time of NDA submission. Under priority review, the overall regulatory review time frame is substantially shortened. By the end of December 2025, a total of 189 NDA submissions for anti-tumor drugs had been granted priority review. Of these, 86 qualified based on prior BTD designation, and 92 were approved under the conditional approval pathway. The median review duration for priority review-designated candidates was 143 working days. In comparison, 317 NDA submissions for anti-tumor drugs did not receive priority review, with a median review duration of 232 working days. The integrated use of BTD, Priority Review, and Conditional Approval forms a tiered and differentiated review system tailored to drugs at different developmental stages, thereby substantially enhancing the efficiency of innovative drug development.

In recent years, the NMPA has implemented targeted policies to allocate the review resources to priority innovation areas and guide industrial development through optimized regulatory pathways. In 2024, the NMPA launched the Pilot Program on Optimizing the Review and Approval of Clinical Trials for Innovative Drugs [6], enabling innovative drug IND applications to be reviewed within 30 working days. The pilot covered 30 applications, including 13 anti-tumor drugs. Building on pilot experience, the *Announcement on Optimizing of the Review and Approval Process for Clinical Trials of Innovative Drugs* issued in 2025 [7] stipulates that eligible applications for INDs involving innovative drugs or global Phase III clinical trials under global simultaneous development shall be reviewed within 30 working days.

## Reshaping the Landscape of Oncology Therapeutics

China lagged in the development of anti-tumor drugs. Multinational companies lacked incentives to launch novel, highly effective anti-tumor drugs in China, resulting in substantial delays in market entry. For instance, Blinatumomab (Blincyto), the first bispecific antibody approved globally for acute lymphoblastic leukemia, was first approved by the U.S. Food and Drug Administration in 2014 [8]. However, its NDA was not submitted in China until 2019. The approval and accessibility gaps deprived Chinese patients of access to the latest therapies and created inconsistencies between Chinese clinical practices and global standards, further impeding global simultaneous drug development programs.

Institutions and policy innovation have removed the barriers to the introduction of drugs developed outside China, narrowing the gap in drug accessibility between China and developed countries. From 2021 to 2025, the CDE approved 154 anti-tumor drug NDA submissions for imported drugs (defined as a medicinal product produced outside mainland China). Among these, 55 were submitted under the global simultaneous development model (at the time of NDA submission, the corresponding indication had not yet been approved overseas), including 10 innovative drugs that were previously unapproved in any country.

Enhanced regulatory review capacity has driven improvements in clinical practice. Taking breast cancer as an example, novel drugs with new mechanisms or new targets for breast cancer, such as human epidermal growth factor receptor 2 (HER2) antibody–drug conjugate (ADC) and phosphoinositide 3-kinase α (PI3Kα) inhibitors, have all been approved in China. The HER2 ADC drug trastuzumab deruxtecan has been successively approved for indications in HER2-positive breast cancer [9], HER2-low breast cancer [10], and HER2-ultralow breast cancer [11]. Among these, the indications for HER2-low and HER2-ultralow breast cancer achieved global simultaneous approval. Inavolisib, a highly specific *PI3Kα* inhibitor, was first approved by the U.S. Food and Drug Administration [12] and subsequently in China via a prioritized review and approval pathway [13], addressing the unmet need for precision therapy for HR^+^/HER2*^−^* advanced breast cancer harboring phosphatidylinositol-4,5-bisphosphate 3-kinase catalytic subunit α (*PIK3CA*) mutations.

The favorable innovation ecosystem fostered through regulatory reforms has strongly propelled the advancement of domestic innovative drug development. The R&D strategies of anti-tumor drug development have evolved from fast-follow to best-in-class and further to first-in-class innovation. Becotatug vedotin (MRG03), the world’s first epidermal growth factor receptor (*EGFR*)-targeted ADC, was approved by the NMPA in October 2025 for recurrent or metastatic nasopharyngeal cancer [14]. Cadonilimab, the world’s first bispecific antibody targeting programmed cell death protein 1 and cytotoxic T lymphocyte-associated antigen 4 (CTLA-4), received marketing approval in China in June 2025 for the treatment of recurrent or metastatic cervical cancer [15]. Ivonescimab was approved in May 2024 in China for the treatment of non-small cell lung cancer [16], becoming the world’s first bispecific antibody targeting programmed cell death protein 1 and vascular endothelial growth factor. In addition, ivonescimab significantly improved progression-free survival compared with pembrolizumab in previously untreated patients with advanced programmed death-ligand 1-positive non-small cell lung cancer (HARMONi-2, NCT05499390) [17].

Ivonescimab is now established as a standard therapeutic option in Chinese clinical practice. Chinese clinical practice may once again diverge from the global norms, yet this time, China’s standard of care demonstrates superior efficacy. This shift will create new opportunities for global drug development, deliver greater benefits to patients worldwide, and pose new challenges for regulators.

## Conclusion

From reliance on generic drug manufacturing to the advancement of first-in-class innovative drugs, China’s regulatory reforms have driven a historic transition in the development of anti-tumor drugs. Nonetheless, deeper and more fundamental reforms remain essential. Efficient R&D and regulatory processes have elevated China from the position of a follower to a global competitor, and even a pacemaker in certain areas. However, greater emphasis on original innovation is still required.

Notable breakthroughs in bispecific antibodies and ADCs have largely been built on established platform technology and known targets or mechanisms. Additional efforts are needed for the discovery of novel targets and new mechanisms of action, as well as for the effective translation of basic research advances into drug development.

Going forward, China will remain committed to reform and opening up, keep abreast of global advances in drug innovation, and strengthen the research on regulatory science. Building on the benefits from reforms, Chinese authorities will continue to refine the innovation ecosystem to provide better technical support and service for the R&D of innovative drugs. Concurrently, efforts will be made to promote cross-disciplinary research and collaboration, integrate basic research and clinical needs in an organic way, and identify and characterize novel targets with global relevance. Ultimately, these endeavors will translate into more novel therapies that are safe, efficacious, and accessible, bringing benefit to the health of the people.

## Data Availability

The datasets used in the current study are available from the corresponding authors upon reasonable request.
